# The evolution of health status and chronic conditions in Catalonia, 1994-2006: the paradox of health revisited using the Blinder - Oaxaca decomposition

**DOI:** 10.1186/1472-6963-11-116

**Published:** 2011-05-23

**Authors:** Anna García-Altés, Jaime Pinilla, Vicente Ortún

**Affiliations:** 1 Agència de Salut Pública de Barcelona, Spain; 2 Fundación Instituto de Investigación en Servicios de Salud, Spain; 3 CIBER Epidemiología y Salud Pública (CIBERESP), Spain; 4 Departamento de Métodos Cuantitativos en Economía y Gestión, Universidad de Las Palmas de Gran Canaria, Spain; 5 Departament d'Economia i Empresa, Universitat Pompeu Fabra, Spain; 6 Centre de Recerca en Economia i Salut, Universitat Pompeu Fabra, Spain

**Keywords:** health status, chronic conditions, prevalence, severity, Blinder - Oaxaca decomposition

## Abstract

**Background:**

The paradox of health refers to the improvement in objective measures of health and the increase in the reported prevalence of chronic conditions. The objective of this paper is to test the paradox of health in Catalonia from 1994 to 2006.

**Methods:**

Longitudinal cross-sectional study using the Catalonia Health Interview Survey of 1994 and 2006. The approach used was the three-fold Blinder - Oaxaca decomposition, separating the part of the differential in mean visual analogue scale value (VAS) due to group differences in the predictors (prevalence effect), due to differences in the coefficients (severity effect), and an interaction term. Variables included were the VAS value, education level, labour status, marital status, all common chronic conditions over the two cross-sections, and a variable for non-common chronic conditions and other conditions. Sample weights have been applied.

**Results:**

Results show that there is an increase in mean VAS for men aged 15-44, and a decrease in mean VAS for women aged 65-74 and 75 and more. The increase in mean VAS for men aged 15-44 could be explained by a decrease in the severity effect, which offsets the increase in the prevalence effect. The decrease in mean VAS for women aged 65-74 and 75 and more could be explained by an increase in the prevalence effect, which does not offset the decrease in the severity effect.

**Conclusions:**

The results of the present analysis corroborate the paradox of health hypothesis for the population of Catalonia, and highlight the need to be careful when measuring population health over time, as well as their usefulness to detect population's perceptions.

## Background

The assessment of the health status of populations has important consequences in health policy. Arguments in favour of limiting health spending due to its low marginal productivity have no value if health spending is not assessed in parallel with changes in population health status as a consequence of this spending. Along with the assessment of benefits in terms of changes in health status it is also relevant to analyze allocation of resources among health programs, as is the case of a standard economic evaluation. Finally, health status has also influence in other individual behaviours, such as labour market decisions, and investment in health [1-4].

Data shows that objective measures of health have been continuously improving in developed countries. Catalonia is one of the 17 Autonomous Communities of Spain situated in the northeast of Spain, with an area of 31,895 km^2^. Catalonia, as the rest of Spain, has a National Health Service financed mainly by taxes, which provides universal and free healthcare coverage. Over the period 1994-2006, population grew from 6.09 million inhabitants to 7.13 million; the percentage of foreign population increased (1.61% to 12.81%), as well as the percentage of population over 65 years old (15.70% to 16.39%). Life expectancy increased for men from 74.8 years in 1994 to 78.2 years in 2006; for women, life expectancy was 82.0 years in 1994 and went up to 84.5 years in 2006 [5]. However, there has also been an increase in the reported prevalence of chronic conditions, and doctor consultations. This fact (improvement in objective measures of health and increase in the reported prevalence of chronic conditions) has been coined as "the paradox of health", and several factors have been suggested to explain it: the decrease in mortality due to acute diseases, increase in the awareness of bodily symptoms, changes over time on diagnosing illness, commercialization of health, and increase in the expectation of being cured [6-8].

The consequences of the increase in the reporting of chronic conditions are many. In particular, chronic conditions have a direct impact when measuring the health status of populations using EQ-5D: it leads to more severe health states, worse health status and decreases in health capital estimation. In Catalonia were this instrument has been used, it has been reported that the health of the Catalan population has worsened from 1994 to 2006, especially for women, contradicting the observed improvements in life expectancy [9]. Also for Sweden, results for 1980/81 and 1996/97 showed considerable health gains for older people and small or non-existent gains for younger women [10].

The objective of this paper is to test "the paradox of health" in Catalonia, analysing health status changes from 1994 to 2006, and decomposing the estimated differences into three components: a "prevalence effect" of chronic conditions, a "severity effect", and an interaction due to simultaneous differences in prevalence and severity of chronic conditions between these years.

## Methods

A longitudinal cross-sectional study was carried out using the Catalonia Health Survey of 1994 and 2006 (i.e. not panel data). The Catalonia Health Survey collects, by means of direct personal interviews, demographic variables, self-assessed health status, chronic diseases, perceived morbidity, utilization of healthcare services and drugs, health-related lifestyles, and healthcare coverage. The study population corresponded to the survey sample of the interviews, i.e. adults in Catalonia excluding those institutionalized, and the unit of analysis corresponding to the individuals that responded to the surveys of 1994 (n = 15,000), and 2006 (n = 15,553).

To test "the paradox of health", we decomposed the change in health status from 1994 to 2006 for every sex and age group (15-44, 45-64, 65-74, and 75 years old and more) into three components: a "prevalence effect" due to year differences in the distribution of chronic conditions, a "severity effect" due to year differences in the impact of these conditions on population health, and an interaction due to simultaneous differences in prevalence and severity between the two years. Health status was measured using the visual analogue scale (VAS) of the EQ-5D instrument that the survey includes to measure quality of life and utilities [11]. We controlled for socio-demographic variables that may be related with health status (i.e. education level, labour status, marital status). The variables included in the model and their categorizations are:

- Health status: VAS value, measured from 0 ("worst imaginable health state") to 100 ("best imaginable health state").

- Education level, coded 1 to 4: non studies, primary studies, secondary studies, university studies.

- Labour status, coded 1 to 5: employed, unemployed, home work, retired, student.

- Marital status, coded 1 to 4: single, married, divorced, widowed.

- All common chronic conditions over the two cross-sections. Respondents are asked: "Do you currently have or did your doctor tell you that you had any of the following chronic conditions?" The common conditions were hypertension, varicose veins, osteoarthritis, allergy, bronchitis, diabetes, duodenal ulcer, high cholesterol, cataracts, constipation, nervous problems-depression, embolism, cardiac problems, asthma, skin problems (for each: "no = 0", "yes = 1"). Prostate or urinary problems, despite being common, were not included because of collinearity problems.

- A variable for non-common chronic conditions and other conditions. Non-common conditions over the two cross-sections were migraine, back pain, blood circulation problems, moraines, thyroid problems, cancer tumor, anemia, heart attack, cervical pain, osteoporosis, incontinence problems. The construction of the variable also included "other conditions", filled in both surveys as free text. The composite variable was coded "no = 0", "yes = 1".

For every sex and age group, a linear regression was run to relate VAS values with all demographic and chronic condition variables. We used "non studies", "employed", and "married", as reference categories of the categorical variables. Variables with statistically significant coefficients were included in the decomposition. The decomposition approach we adopted is the well-known one from labour economics associated with Blinder - Oaxaca [12,13], that has become a standard technique for decomposing gaps in outcomes such as wages between different population groups (according to sex, race, or any other characteristic), although we used the three-fold decomposition variant.

Given two groups (1994 and 2006), an outcome variable (mean VAS), and a set of predictors, the question is how much of the mean outcome difference R, where:

is accounted for by group differences in the predictors. Based on the linear model:

where Y_i _refers to the outcome (mean VAS) for each group (1994 and 2006), X'_i _is a vector of characteristics (sociodemographic and chronic conditions), β_i _is the associated parameter vector, and ε_i _is an error term. The three-fold Blinder - Oaxaca decomposition breaks down the difference between 2006 and 1994 in the following way [14]:

where E(vas_2006_) is the predicted mean VAS for 2006, X'_2006 _is the mean vector of characteristics for 2006 that determine VAS, and β_2006 _is the vector of estimated returns to VAS determinants for 2006 (likewise for 1994). The first summand of the right hand side accounts for the part of the differential that is due to group differences in the predictors ("prevalence effect"), the second measures the contribution of differences in the coefficients ("severity effect"), while the third summand is an interaction term accounting for the fact that differences in endowments and coefficients exist simultaneously between the two groups.

Blinder - Oaxaca decompositions have typically been carried out using linear regression models owing the attractive property that such models fit exactly at the mean of the sample, but the approach has also been used for binary, ordered and count models [15,16]. The analysis above assumes that the dependent variable is continuous. In this paper, the dependent variable is an ordered categorical variable, albeit with quite a high number of categories (from 0 to 100). While Blinder - Oaxaca type decompositions can be carried out with ordered response models, non-linear approximations make impossible to estimate the contribution of each individual variable to the prevalence and severity effects.

Although VAS data was not normally distributed, we choose to model untransformed data. Transformations, such as log or square root are often proposed, but provide estimates with difference on a scale not relevant to policy making interpretation [17]. Moreover, the assumption of normality is primarily a convenience for the purpose of statistical inference; when this assumption fails to hold, the estimates of fixed and random parameters will still be consistent, though the standard error estimates will be inconsistent in small samples [18].

So, we estimated and carried out the decomposition assuming that VAS is a continuous variable and employed the linear specification above. To check for the consistency of this procedure we compared the results obtained using the linear Blinder - Oaxaca decomposition and the non-linear one (ordered probit specification). Since the number of values was different from 1994 to 2006 (i.e., the scale has the same 0 to 100 values, but people responded to a different number of categories in both years), we recoded VAS values into 20 groups (grouping VAS values in groups of 5), and run both the ordered probit specification and the linear one.

Sample weights have been applied. Significance level was set at p = 0.05. All analyses were performed using Stata 10.1, using the oaxaca command.

## Results

Table 1 shows the frequencies of the variables included in the model for every sex and age group. The sex distribution is similar in the two surveys and the age distribution changes slightly, showing an increase from 1994 to 2006 in the 15-44 group, and in the 75 years and older age -especially for women- owing to the changes in the demography of Catalonia. There is also an increase of men and women undertaking secondary and university studies, and an increase in the proportion of people employed between these years.

**Table 1:** Socio-demographic characteristics by sex and age group.

	Men 15-44		Men 45-64		Men 65-74		Men 75 & more	
	**1994**	**2006**	**1994**	**2006**	**1994**	**2006**	**1994**	**2006**

N	3,030	4,097	1,747	2,240	715	801	380	743

Age group	52.83	56.24	29.73	27.69	11.42	8.86	6.02	7.21

Married	41.15	39.22	88.76	82.61	88.28	86.18	71.14	76.94

Single	58.06	58.34	7.11	9.28	4.19	6.32	3.33	5.45

Divorced	0.76	2.38	2.03	6.09	1.30	1.93	0.82	0.65

Widow	0.03	0.06	2.10	2.02	6.23	5.57	24.71	16.96

Employed	61.84	80.57	67.70	77.92	2.67	3.60	0.52	0.74

Unemployed	13.55	4.81	10.69	7.07	0.00	0.71	0.00	0.12

Home work	0.00	0.06	0.00	0.15	0.00	0.17	0.00	0.43

Retired	0.11	0.28	12.09	8.45	92.23	91.03	95.33	93.19

Student	24.5	14.28	9.52	6.41	5.10	4.49	4.15	5.52

Non studies	1.38	2.56	15.69	9.49	21.82	30.76	32.75	42.65

Primary studies	49.41	38.35	61.76	48.83	64.59	48.05	56.39	41.08

Secondary studies	37.64	41.15	11.40	23.96	8.12	11.98	5.51	9.87

University studies	11.57	17.94	11.15	17.72	5.47	9.21	5.35	6.40

	**Women 15-44**		**Women 45-64**		**Women 65-74**		**Women 75 & more**	

	**1994**	**2006**	**1994**	**2006**	**1994**	**2006**	**1994**	**2006**

N	3,229	3,797	1,935	2,226	871	902	660	1,120

Age group	49.29	50.52	28.76	27.70	12.65	10.01	9.30	11.77

Married	50.53	47.10	82.06	75.78	61.94	65.23	20.40	30.28

Single	46.28	47.63	5.47	7.95	7.33	4.64	7.75	8.31

Divorced	2.93	4.88	3.95	9.54	1.26	3.27	1.29	0.68

Widow	0.26	0.39	8.52	6.73	29.47	26.86	70.56	60.73

Employed	43.55	69.80	27.95	52.44	0.96	2.18	0.13	0.47

Unemployed	12.95	6.65	3.68	5.45	0.08	0.48	0.00	0.69

Home work	20.59	9.73	59.13	32.65	51.53	46.07	42.06	43.52

Retired	0.00	0.11	4.12	4.58	40.79	46.78	50.51	49.26

Student	22.91	13.71	5.12	4.88	6.64	4.49	7.30	6.06

Non studies	2.08	2.02	21.87	15.29	34.86	45.89	46.55	59.75

Primary studies	47.18	32.80	64.95	50.89	58.75	43.59	49.82	32.77

Secondary studies	37.05	40.14	7.88	20.29	4.72	7.24	2.42	4.35

University studies	13.69	25.04	5.30	13.53	1.67	3.28	1.21	3.13

Catalonia, 1994-2006.

**Figure 1:**
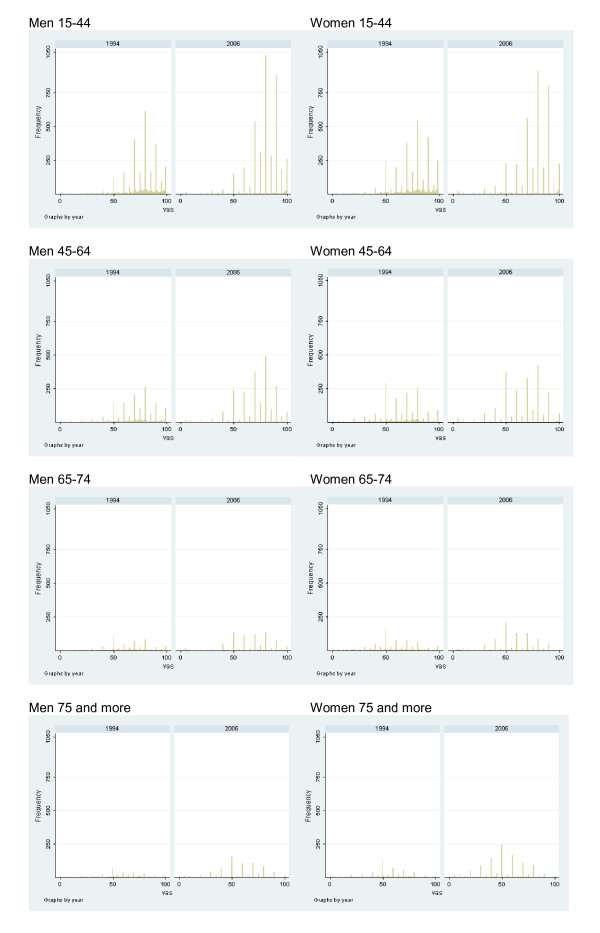
Frequency distribution of VAS by sex and age group. Catalonia, 1994-2006

Table 2 shows the prevalence of chronic conditions by sex and age group. Both men and women tend to declare more chronic conditions through time, especially nervous problems-depression, high cholesterol, and cataracts. A few conditions have decreased in prevalence, such as osteoarthritis in both sexes and bronchitis among men. Table 3 shows mean VAS values by sex and age group. VAS has statistically significantly increased for men aged 15-44 years (from 77.57 in 1994 to 79.02 in 2006) and has decreased for women aged 65-74 years (58.92 to 55.13) and 75 and more years old (56.32 to 50.43); Figure 1 shows graphically the change over time in the distribution of VAS by sex and age group.

**Table 2:** Prevalence of chronic conditions by sex and age group.

	Men 15-44		Men 45-64		Men 65-74		Men 75 & more	
	**1994**	**2006**	**1994**	**2006**	**1994**	**2006**	**1994**	**2006**

Hypertension	4.71	5.77	19.35	28.93	30.16	46.99	27.97	40.11

Cardiac problems	1.60	2.18	7.74	7.94	16.56	20.41	21.44	29.65

Varicose veins	2.55	3.38	8.00	13.46	10.78	15.71	13.59	21.01

Osteoarthritis	10.51	3.07	34.34	20.92	45.84	42.26	42.98	49.91

Allergy	13.58	16.35	8.27	13.64	6.62	11.05	8.34	11.08

Asthma	3.89	4.84	4.29	4.37	7.81	7.86	7.31	12.14

Bronchitis	3.91	3.21	9.58	7.36	20.20	17.37	20.42	20.52

Diabetes	0.92	0.97	6.48	7.81	13.17	16.49	8.42	17.14

Duodenal ulcer	3.54	2.97	10.52	9.46	13.19	13.49	9.65	14.15

Cholesterol	4.27	6.39	16.72	25.65	14.09	26.16	7.95	21.75

Cataract	0.33	0.52	2.91	3.58	12.07	18.50	25.88	40.36

Skin problems	4.71	4.87	4.69	7.26	4.82	8.63	6.48	14.06

Constipation	1.44	2.06	3.59	3.84	7.17	8.76	9.01	18.08

Nervous problems	4.61	7.42	8.22	14.62	7.65	15.89	8.78	18.66

Embolia	0.30	4.00	1.70	1.75	3.90	4.67	7.72	8.93

Non common	22.51	41.52	35.70	61.24	43.62	73.04	60.33	77.43

	**Women 15-44**		**Women 45-64**		**Women 65-74**		**Women 75 & more**	

	**1994**	**2006**	**1994**	**2006**	**1994**	**2006**	**1994**	**2006**

Hypertension	3.66	5.32	27.37	24.99	42.15	51.42	42.69	52.73

Cardiac problems	1.89	2.06	6.72	6.57	15.14	19.45	21.81	26.55

Varicose veins	8.64	17.84	63.97	40.41	61.97	45.31	74.94	42.23

Osteoarthritis	15.34	4.97	57.21	41.40	73.40	73.51	66.28	76.14

Allergy	17.32	17.92	17.49	17.60	16.13	19.75	10.77	14.77

Asthma	3.41	5.86	4.57	5.84	7.16	10.12	8.39	9.25

Bronchitis	2.82	3.75	4.98	5.17	9.84	11.16	11.93	10.00

Diabetes	0.73	1.08	6.98	6.95	12.99	17.43	11.23	17.95

Duodenal ulcer	1.96	2.47	6.56	6.45	7.67	9.28	6.51	10.01

Cholesterol	3.22	4.28	16.57	23.53	2.48	30.41	14.28	30.18

Cataract	1.00	0.37	3.71	4.97	16.60	26.65	34.84	52.66

Skin problems	5.46	7.25	5.21	9.02	8.77	8.78	8.19	11.56

Constipation	8.50	8.36	16.24	17.42	18.13	20.65	23.85	27.34

Nervous problems	9.23	15.16	22.25	30.74	22.02	36.76	13.96	33.65

Embolia	0.32	0.35	1.36	0.92	3.38	5.04	6.30	7.50

Non common	25.70	58.35	40.82	79.68	48.16	88.50	56.41	87.94

Catalonia, 1994-2006.

**Table 3:** Mean VAS values by sex and age group.

Sex and age group	1994	2006	Difference	p-value
Men 15-44	77.57	79.02	1.45	0.000

Men 45-64	71.24	70.58	-0.66	0.315

Men 65-74	63.96	65.20	1.24	0.293

Men 75 & more	59.59	58.67	-0.92	0.523

Women 15-44	75.74	76.44	0.7	0.121

Women 45-64	64.74	65.46	0.72	0.307

Women 65-74	58.92	55.13	-3.79	0.001

Women 75 & more	56.32	50.43	-5.89	0.000

Catalonia, 1994-2006.

Regarding the results of the Blinder - Oaxaca decomposition, Table 4 shows the contribution of each variable to the prevalence effect, the severity effect and their interaction to the gap in VAS. For men aged 15-44 years, if the prevalence of chronic conditions in 1994 would be that of 2006, mean VAS would be lower (-0.396), corresponding to the increase in prevalence of 2006. On the other side, if the severity of chronic conditions in 1994 would be that of 2006, mean VAS would be higher (1.714). So, the increase of 1.504 in mean VAS is mainly due to a decrease in the severity effect. Looking at the specific variables, there is a positive contribution to the prevalence effect of osteoarthritis (it decreases from 1994 to 2006), and a negative contribution of nervous problems-depression and non common chronic conditions (both increase from 1994 to 2006). The severity effect is driven by the positive contribution of nervous problems-depression, and the negative contribution of osteoarthritis.

**Table 4:** Detailed results of the Blinder-Oaxaca decomposition.

	Men 15 - 44			Women 65 - 74			Women 75 & more		
	**Coef**.	**Std. Er**.	**P > |t|**	**Coef**.	**Std. Er**.	**P > |t|**	**Coef**.	**Std. Er**.	**P > |t|**

Prediction_06	79.049	0.264	0.000	54.873	0.833	0.000	50.644	0.828	0.000

Prediction_94	77.545	0.299	0.000	58.928	0.742	0.000	56.277	0.828	0.000

Difference	1.504	0.399	0.000	-4.055	1.115	0.000	-5.632	1.170	0.000

**Prevalence effect**									

Studies level	0.067	0.090	0.457	-0.238	0.318	0.455	-0.496	0.342	0.147

Labour status	0.253	0.117	0.030	0.306	0.168	0.068	-0.024	0.164	0.885

Marital status	-	-	-	-	-	-	-0.382	0.239	0.110

Hypertension	-0.018	0.019	0.347	-	-	-	-	-	-

Varicose veins	0.002	0.015	0.882	-	-	-	-	-	-

Osteoarthritis	0.410	0.096	0.000	-0.083	0.217	0.702	-0.781	0.267	0.003

Bronchitis	0.042	0.034	0.221	-0.078	0.123	0.522	-	-	-

Diabetes	-0.001	0.004	0.768	-0.199	0.135	0.139	-0.336	0.186	0.071

Duodenal ulcer	0.035	0.032	0.262	-0.099	0.099	0.319	-	-	-

Cholesterol	-0.035	0.036	0.321	-	-	-	-	-	-

Constipation	-	-	-	-	-	-	-0.223	0.173	0.196

Nervous problems	-0.401	0.107	0.000	-1.019	0.289	0.000	-1.455	0.479	0.002

Skin problems	-0.004	0.015	0.787	-	-	-	-	-	-

Embolia	-	-	-	-0.094	0.100	0.343	-	-	-

Cardiac problems	-	-	-	-	-	-	-0.151	0.113	0.184

Asthma	-	-	-	-	-	-	-0.065	0.098	0.504

Non common	-0.745	0.144	0.000	-1.489	0.548	0.007	-1.698	0.476	0.000

Total	-0.396	0.268	0.139	-2.990	0.841	0.000	-5.612	0.887	0.000

**Severity effect**									

Studies level	-1.275	1.021	0.212	3.225	1.514	0.033	-3.353	2.700	0.214

Labour status	7.032	2.121	0.001	6.968	1.825	0.000	-8.431	3.076	0.006

Marital status	-	-	-	-	-	-	-3.044	2.549	0.233

Hypertension	-0.044	0.086	0.609	-	-	-	-	-	-

Varicose veins	-0.118	0.062	0.059	-	-	-	-	-	-

Osteoarthritis	-0.541	0.263	0.040	1.525	1.701	0.370	0.827	1.570	0.598

Bronchitis	0.130	0.104	0.210	0.139	0.346	0.687	-	-	-

Diabetes	-0.036	0.032	0.269	-0.035	0.377	0.926	0.324	0.346	0.350

Duodenal ulcer	0.098	0.089	0.273	0.288	0.265	0.277	-	-	-

Cholesterol	-0.072	0.084	0.386	-	-	-	-	-	-

Constipation	-	-	-	-	-	-	0.887	0.580	0.126

Nervous problems	0.277	0.106	0.009	-0.733	0.487	0.132	-0.488	0.377	0.196

Skin problems	0.025	0.091	0.782	-	-	-	-	-	-

Embolia	-	-	-	-0.238	0.198	0.229	-	-	-

Cardiac problems	-	-	-	-	-	-	-0.210	0.491	0.668

Asthma	-	-	-	-	-	-	-0.239	0.311	0.443

Non common	-0.170	0.198	0.389	-2.984	1.156	0.010	-0.774	1.526	0.612

Constant	-3.592	2.407	0.136	-6.187	2.893	0.033	14.749	5.256	0.005

Total	1.714	0.458	0.000	1.966	1.217	0.106	0.248	1.305	0.849

**Interaction**									

Studies level	0.071	0.104	0.496	-0.088	0.315	0.781	-0.244	0.450	0.589

Labour status	-0.158	0.191	0.407	0.147	0.217	0.500	0.213	0.139	0.126

Marital status	-	-	-	-	-	-	0.571	0.339	0.093

Hypertension	-0.011	0.021	0.622	-	-	-	-	-	-

Varicose veins	-0.043	0.031	0.171	-	-	-	-	-	-

Osteoarthritis	0.383	0.188	0.042	0.020	0.056	0.724	0.124	0.237	0.602

Bronchitis	-0.023	0.025	0.363	0.016	0.047	0.731	-	-	-

Diabetes	-0.004	0.012	0.718	-0.012	0.130	0.926	0.181	0.201	0.368

Duodenal ulcer	-0.016	0.020	0.422	0.069	0.087	0.426	-	-	-

Cholesterol	-0.035	0.042	0.400	-	-	-	-	-	-

Constipation	-	-	-	-	-	-	0.139	0.134	0.301

Nervous problems	0.167	0.073	0.022	-0.511	0.348	0.142	-0.734	0.567	0.196

Skin problems	0.001	0.005	0.846	-	-	-	-	-	-

Embolia	-	-	-	-0.118	0.125	0.344	-	-	-

Cardiac problems	-	-	-	-	-	-	-0.050	0.120	0.675

Asthma	-	-	-	-	-	-	-0.035	0.067	0.603

Non common	-0.146	0.170	0.389	-2.549	0.993	0.010	-0.432	0.852	0.612

Total	0.186	0.343	0.588	-3.026	1.067	0.005	-0.268	1.116	0.810

Catalonia, 1994-2006.

For women aged 65-74 years, if the prevalence of chronic conditions in 1994 would be that of 2006, mean VAS would be lower (-2.990), corresponding to the increase in prevalence of 2006. On the other side, if the severity of chronic conditions in 1994 would be that of 2006, mean VAS would be higher (1.966). So, the decrease in mean VAS (-4.055) is due to an increase in the prevalence effect. Regarding the specific variables, there is a negative contribution to the prevalence effect of nervous problems-depression and non common chronic conditions (both increase from 1994 to 2006), and a negative contribution to the severity effect of non common chronic conditions.

**Figure 2:**
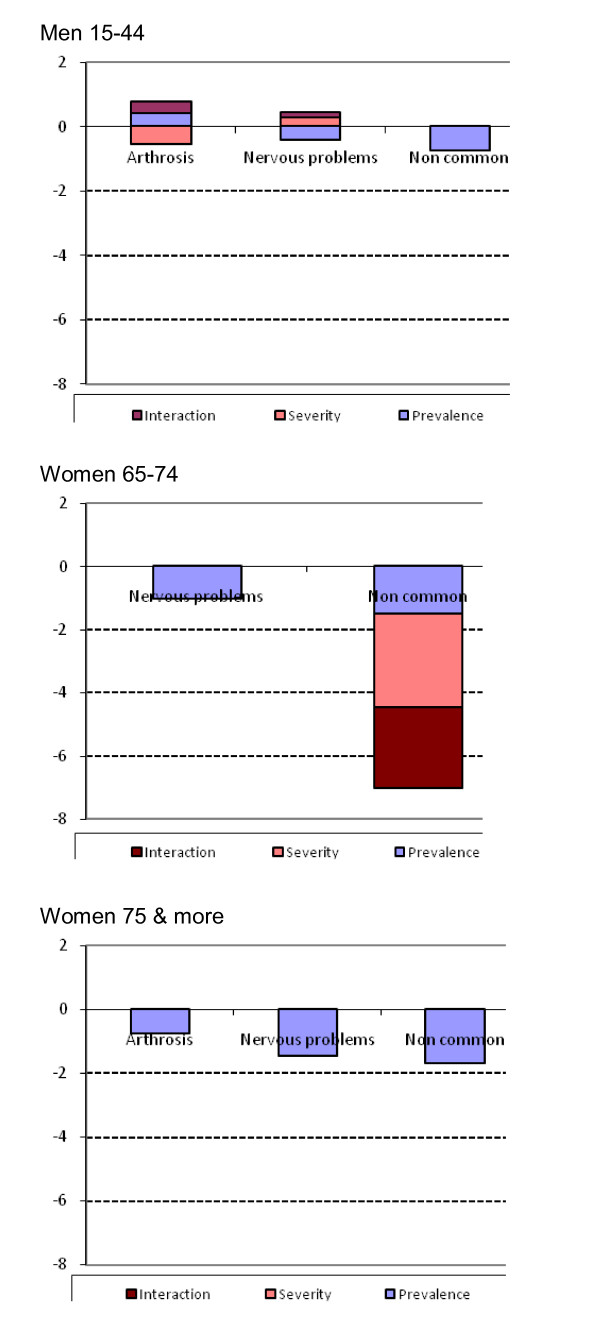
Contribution to the gap in VAS by each variable to the prevalence, severity and interaction effects. Catalonia, 1994-2006.

For women aged 75 years and more, if the prevalence of chronic conditions in 1994 would be that of 2006, mean VAS would be lower (-5.612), corresponding to the increase in prevalence of 2006. On the other side, if the severity of chronic conditions in 1994 would be that of 2006, mean VAS would be higher (0.248). So, the decrease in mean VAS (-5.632) is due to an increase in the prevalence effect. Regarding the specific variables, there is a positive contribution of osteoarthritis (it decreases its prevalence from 1994 to 2006) and a negative contribution to the prevalence effect of nervous problems-depression and non common chronic conditions (both increase their prevalence from 1994 to 2006). Figure 2 shows graphically the contribution of each variable to the prevalence and severity effect, and the interaction between both.

Table 5 shows the gap in VAS and the contribution to the gap by differences in prevalence and severity effects, and the interaction among both, using the linear model and the non-linear one, once the VAS variable has been recoded. The qualitative similarity of the obtained results reassures us in the little influence of the linearity assumption on the results of the paper.

**Table 5:** Gap in VAS, and contribution to the gap by prevalence, severity and interaction effects.

	Linear model	Ordered probit model
**Men 15-44**		

Gap in VAS	0.196	0.186

Prevalence effect	-0.082	-0.102

Severity effect	0.239	0.030

Interaction	0.038	-0.012

**Women 65-74**		

Difference	-0.902	-0.664

Prevalence effect	-0.575	-0.699

Severity effect	0.305	0.372

Interaction	-0.632	-0.646

**Women 75 & more**		

Difference	-1.205	-1.182

Prevalence effect	-1.117	-1.189

Severity effect	-0.839	-0.088

Interaction	-0.049	-0.905

Catalonia, 1994-2006.

## Discussion

Results of the analysis show that there is an increase in mean VAS for men aged 15-44 years, and a decrease in mean VAS for women aged 65-74 years and 75 years and more. The increase in mean VAS for men aged 15-44 years could be explained by a decrease in the severity effect, which offsets the increase in the prevalence effect. In particular, osteoarthritis increases prevalence and decreases severity; and nervous problems-depression increase its prevalence and severity. The decrease in mean VAS for women aged 65-74 years and 75 years and more could be explained by an increase in the prevalence effect, which does not offset the decrease in the severity effect. The increase in prevalence is especially notorious for osteoarthritis, nervous problems-depression and non common conditions.

For the three age and sex groups with statistically significant changes in mean VAS, there has been an increase in the prevalence effect and a decrease in the severity effect. Following the paradox of health, despite the increase in the prevalence of chronic conditions, from 1994 to 2006 there has been an improvement in health status for men aged 15-44 years, both measured as self-perceived health (VAS) and as life expectancy. The improvement in their self-perceived health is due to a decrease in the severity effect that offsets the increase in the prevalence effect of chronic conditions. For women aged 65-74 years and 75 years and more, although there is an improvement of their life expectancy, the prevalence of chronic conditions increases, and their self-perceived health decrease due to an increase in the prevalence effect, which offsets the decrease in the severity effect. Differences among younger men and older women may result from the positive and statistically significant effect of labour status on the prevalence and the severity effect for men aged 15-44 years. These results are consistent with previous research on male and female differences on self-assessed health and chronic conditions [8].

As previously mentioned, several factors could help us to explain the increase in the prevalence effect and the decrease in the severity effect of chronic conditions in self-reported health: the decrease in mortality due to acute diseases; the increase in the awareness of bodily symptoms; the varying idealized states of health and the willingness of an individual to acknowledge sickness; the changes over time in diagnosing illness (people are screened more often, and thresholds are lower); the commercialization of health; the increase in the expectation of being cured; the phenomenon of adaptation to illness and even the conscious misreporting of morbidity to achieve other goals (labour participation and government benefits) [6,19-21].

In particular, it is worth mentioning the considerable increase in the prevalence of some chronic conditions over time, like nervous problems-depression, that could be related to the increase in the availability of drugs for their treatment, and cataracts, which could be explained by the increase in their diagnosis and treatment by means of surgery. On the contrary, osteoarthritis has decreased, probably due to the fact that the questionnaire in 2006 included other related conditions, such as backache and osteoporosis. Bronchitis has also decreased among men, consistent with development of the smoking epidemic in the last century. Also, as this is not panel data, the increase in the diagnosis of chronic conditions could be at the expense of adding less severe individuals to the sample in the most recent cohorts.

The analysis performed is an application of the Blinder - Oaxaca decomposition to health data. The Blinder - Oaxaca decomposition is widely used to identify and quantify the separate contributions of group differences in measurable characteristics, such as education, experience, marital status, etc., to racial and gender gaps in outcomes. Although this methodology has been extensively applied in labour economics to analyzed wage differentials [13,14,22], its application to health is much less frequent [23-26]. Regarding the use of a linear approximation, the qualitative similarity of the obtained results reassures us that the influence of the linearity assumption on the results of the paper is not that significant.

The work is not absent of limitations. The main one would be the use of self-declared information, both regarding health status and chronic conditions, although a good correlation of subjective health indicators and final outcome indicators, and even healthcare expenditure, is known [27,28]. Considerable attention has been devoted to the reliability of self-assessed health status and the scope for contamination by measurement error; and there is evidence of reporting bias [29-31]. Different groups (according to age, gender, education, income, language, or personal experience of illness) appear to interpret the questions within their own specific context and therefore use different reference points when responding to the same question, which may invalidate comparisons and measures of health inequality. Whilst the evidence shows mixed results depending on the variable analyzed, the methodology used in this study makes it possible to control for age and sex bias in self-reported health, and the year variable reflects the cohort effect. Additionally, one of the advantages of using VAS as the dependent variable is that we do not carry the effect of changes in cut-points that have been previously described in the literature [30,31], or at least they are minimized since we are using a 0 to 100 points scale.

There is some evidence about misreporting of chronic conditions by respondents' level of education and labour status [20,32]. The effect is minimized here, since the model used controls for education level and labour status. Finally, it has been argued that health interviews have an inherent selection bias, because of the death of some individuals before the interview. The probability of declaring a good or bad health status is conditioned to being alive, so there could be a selective truncation of the distribution function [33].

Also related with the use of health interviews is the comparability of the surveys. We used 1994 survey because it is the oldest one carried out in Catalonia that includes the EQ-5D instrument, and 2006 as it is the most recent. However, the 2006 questionnaire includes many more chronic conditions that the one in 1994. For that reason, we decided to include common conditions in the model, and to create a variable "non common conditions and others", to collapse the rest of the information. Regarding age groups, the ones used are common for the analysis of mortality data, separating younger and older adults, as well as younger and very old; those groups have distinctive epidemiological characteristics, and also different mean VAS, as shown.

## Conclusions

The main implications of the results of our research concern the use of EQ-5D to measure population health over time, and with healthcare policy. As we have previously mentioned, health capital changes over time have been estimated using the same methodology in Catalonia and Sweden, multiplying the amount of life years gained (as a result of increased life expectancy) times the change in quality of life measured by EQ-5D over two cross-sections [9,10]. Respondents to the EQ-5D have reported worse health states over time, coherently with increases in the prevalence of chronic conditions and limitations. The main caveat of this way of proceeding is that utility values used in both studies have been the same in both cross-sections (utility values obtained in 1998 in Spain [34], and for the Swedish work, in 1997 in the UK [35]). So, the worsening in health capital obtained in those studies is a direct consequence of the increase in the prevalence of diseases. The results of our analysis highlight the increase in the prevalence effect, and the decrease in the severity effect, especially among older women, contradicting the results on the decrease of health capital. Using time varying tariffs may take into account the effect of changes in population preferences (i. e decrease in severity effect) across health problems.

Regarding health policy, our results on the increase in the prevalence effect, especially among older women, could be used when planning healthcare services, in particular, to address the health consequences of comorbidity. Perceived illness is a social phenomenon, and self-perceived morbidity provides valuable information on the relevance of disease to the individual. Although the stated worries, observed morbidity and the community's ranking of health problems information is of maximum importance for any health planner concerned with community health and dealing with health priority setting.

## Competing interests

The authors declare that they have no competing interests.

## Authors' contributions

AGA performed the statistical analysis, and drafted the manuscript. JP lead the statistical analysis. VO lead the framing of the study, and the discussion of the results. All authors participated in the conception of the study, and read and approved the final manuscript.

## Pre-publication history

The pre-publication history for this paper can be accessed here:

http://www.biomedcentral.com/1472-6963/11/116/prepub

## References

[B1] Cutler DM Richardson E The value of health: 1970-1990 Am Econ Rev 1998 97 100 AEA Papers and Proceedings

[B2] Sala-i-Martin X Doppelhofer G Miller R Determinants of long-term growth: a Bayesian averaging of classical estimates (BACE) approach Am Econ Rev 2004 45 827 38

[B3] World Health Organization Macroeconomics and health: Investing in health for economic development Report of the Commission on Macroeconomics and Health 2001 Geneva: World Health Organization

[B4] Grossman M On the concept of health capital and the demand for health J Pol Economy 1972 80 2 223 55 10.1086/259880

[B5] IDESCAT Estadística bàsica de Catalunya. Demografia i qualitat de vida. Esperança de vida http://www.idescat.cat/dequavi/?TC=444&V0=2&V1=1 Last accessed: 16/3/10

[B6] Barsky AJ The paradox of health N Engl J Med 1988 318 7 414 8 10.1056/NEJM198802183180705 3340120

[B7] Sen A Health: perception versus observation Br Med J 2002 324 7342 860 1 10.1136/bmj.324.7342.860 PMC1122815 11950717

[B8] Case A Paxson C Sex differences in morbidity and mortality Demography 2005 42 2 189 214 10.1353/dem.2005.0011 15986983

[B9] Zozaya N Oliva J Osuna R Measuring changes in health capital Documento de Trabajo 2005-15 2005 Madrid: FEDEA

[B10] Burström K Johannesson M Diderichsen F The value of the change in health in Sweden 1980/81-1996/97 Health Econ 2003 12 8 637 54 10.1002/hec.754 12898662

[B11] EQ-5D. A standardized instrument for use a a mesure for health outcome http://www.euroqol.org/home.html Last accessed 8/4/11

[B12] Blinder AS Wage discrimination: reduced form and structural estimates J Human Res 1973 8 436 55 10.2307/144855

[B13] Oaxaca R Male-female wage differentials in urban labour markets Int Econ Rev 1973 14 693 709 10.2307/2525981

[B14] Jann BA The Blinder - Oaxaca decomposition for linear regression models The Stata Journal 2008 8 4 453 79

[B15] Fairlie RW An extension of the Blinder - Oaxaca decomposition technique to logit and probit models Center Discussion Paper no. 873 2003 New Haven, CT: Yale University

[B16] Bauer TK Sinning M An extension of the Blinder-Oaxaca decomposition to nonlinear models Advances in Statistical Analysis 2008 92 197 206 10.1007/s10182-008-0056-3

[B17] Manning WG Mullahy J Estimating log models: to transform or not to transform? J Health Econ 2001 20 461 494 10.1016/S0167-6296(01)00086-8 11469231

[B18] Shaw JW Johnson JA Coon SJ US valuation of the EQ-5D health states: development and testing of the D1 valuation model Medical Care 2005 43 3 203 220 10.1097/00005650-200503000-00003 15725977

[B19] Murray CJL Chen LC Understanding morbidity change Pop Develop Rev 1992 18 3 481 503 10.2307/1973655

[B20] Kahneman D Krueger AB Developments in the measurement of subjective well-being J Econ Persp 2006 20 1 3 24 10.1257/089533006776526030

[B21] Baker M Stabile M Deri C What do self-reported, objective measures of health measure? Working paper 8419 2001 Cambridge, MA: National Bureau of Economic Research

[B22] Fairlie RW The absence of the african-american owned business: an analysis of the dynamics of self-employment J Labor Econom 1999 17 1 80 108 10.1086/209914

[B23] Jurges H True health vs response styles: exploring cross-country differences in self-reported health DIW Berlin. Discussion papers 68 2006 Berlin: German Institute for Economic Research 10.1002/hec.1134 16941555

[B24] Madden D Gender differences in mental well-being: a decomposition analysis HEDG working paper 08/08 2008 York: University of York

[B25] O'Donnell O van Doorslaer E Wagstaff A Lindelow M Analyzing health equity using household survey data A guide to techniques and their implementation 2008 Washington, DC: The World Bank

[B26] O'Donnell O van Doorslaer E Wagstaff A Decomposition of inequalities in health and healh care Jones A. Elgar Companion to Health Economics 2006 Chichester: Edward Elgar

[B27] Idler EL Benyamini Y Self-rated health and mortality: a review of twenty-seven community studies J Health Soc Behav 1997 38 1 21 37 10.2307/2955359 9097506

[B28] DeSalvo KB Jones TM Peabody J McDonald J Fihn S Fan V He J Muntner P Health care expenditure prediction with a single item, self-rated health measure Med Care 2009 47 4 440 7 10.1097/MLR.0b013e318190b716 19238099

[B29] Hernández C Quevedo Jones AM Rice N Reporting bias and heterogeneity in self-assessed health. Evidence from the British Household Panel Survey Health, Econometrics and Data Group (HEDG) Working paper 05/04 2005 York: University of York

[B30] Bago d'Uva T O'Donnell O van Doorslaer E Differential health reporting by education level and its impact on the measurement of health inequalities among older Europeans Health, Econometrics and Data Group (HEDG) Working paper 07/28 2008 York: University of York 10.1093/ije/dyn146 PMC2734070 18676985

[B31] Lindeboom M van Doorslaer E Cut-point shift and index shift in self-reported health IZA Discussion Paper No. 1286; Tinbergen Institute Working Paper No. 2003-042/3 2004 Amsterdam: Tinbergen Institute 10.1016/j.jhealeco.2004.01.002 15556237

[B32] Mackenbach JP Looman CWN van der Meer JBW Differences in the misreporting of chronic conditions, by level of education: the effect on inequalities in prevalence rates Am J Public Health 1996 86 706 11 10.2105/AJPH.86.5.706 8629723 PMC1380480

[B33] Heckman J Sample selection bias as a specification error Econometrica 1979 47 153 61 10.2307/1912352

[B34] Badia X Roset M Montserrat S Herdman M Segura A [The Spanish version of EuroQol: a description and its applications. European Quality of Life scale] Med Clin (Barc) 1999 112 Suppl 1 79 85 10618804

[B35] Dolan P Modeling valuations for EuroQol health states Med Care 1997 35 1095 108 10.1097/00005650-199711000-00002 9366889

